# Who’s Behind the Makeup? The Effects of Varying Levels of Cosmetics Application on Perceptions of Facial Attractiveness, Competence, and Sociosexuality

**DOI:** 10.3389/fpsyg.2021.661006

**Published:** 2021-06-17

**Authors:** Erick R. Aguinaldo, Jessie J. Peissig

**Affiliations:** Department of Psychology, California State University, Fullerton, Fullerton, CA, United States

**Keywords:** makeup, cosmetics, sociosexuality, competence, attractiveness, facial attractiveness

## Abstract

Research has demonstrated a positive effect of makeup on facial attractiveness (Cash et al., 1989; Russell, 2003; Etcoff et al., 2011). Makeup has also been found to influence social perceptions (Etcoff et al., 2011; Klatt et al., 2016). While researchers have typically compared faces with makeup to faces without makeup, we propose that perceived effects will differ based on the amount of makeup that is applied. To test the effects of varying levels of makeup on perceived facial attractiveness, competence, and sociosexuality, participants assessed 35 faces with no makeup, light makeup, and heavy makeup; makeup was self-applied by participants, not applied by a makeup artist or the experimenter. Participants rated faces with makeup (either light or heavy) as more competent than those without makeup. In addition, participants rated faces with heavy makeup as significantly higher in attractiveness and sociosexuality than faces with light makeup. These results differ from previous research findings that faces with light makeup (applied by professional makeup artists) are perceived as most attractive. Our results suggest that when makeup is self-applied, faces with heavy makeup are perceived as more attractive and sociosexual than faces with light makeup, and faces with any level of makeup are rated as more competent.

## Introduction

Women in the United States are estimated to spend approximately $3,756 annually on their physical appearance and $225,360 during their lifetime (Haynes, 2018). This high level of spending may be linked to the positive physical and social effects that makeup produces for women. Over the past few decades, many research studies have confirmed that makeup increases facial attractiveness (Cash et al., 1989; Russell, 2003, 2009; Etcoff et al., 2011). Studies have shown that when both male and female participants are asked to rate female faces on attractiveness, faces with makeup are rated as significantly more attractive than those without makeup (Cash et al., 1989; Etcoff et al., 2011). Recent research has gone beyond facial attractiveness and examined how makeup or cosmetics affect peoples’ perceptions of competence, warmth, and trustworthiness (Etcoff et al., 2011; Klatt et al., 2016). Increases in facial attractiveness have been linked to a variety of beneficial social implications.

### Facial Attractiveness

Some have proposed that facial attractiveness is determined solely by culture; however, research suggests a biological basis for attractiveness as well (Berry, 2000). Studies on beauty and attraction across cultures have revealed that people from different cultures typically agree on the attractiveness of faces (Cunningham et al., 1995; Langlois et al., 2000; Rhodes et al., 2001). Additionally, researchers have found that preferences for certain facial characteristics emerge early in development, prior to the periods wherein values and norms from one’s culture are adopted (Geldart et al., 1999; Rubenstein et al., 1999; Slater et al., 2000). These findings provide evidence that contradicts the idea that beauty is based solely on cultural conventions. If this assertion was true, then current findings should indicate significant differences in perceptions of attractiveness across cultures and the development of facial preferences at times where culture begins to influence one’s identity and perspective. Because preferences affect mate choice, Rhodes et al. (2005) suggested that these preferences for certain characteristics may have evolved through sexual selection, whereby traits enhance reproductive success.

Sexual dimorphism refers to feminine traits in female faces and masculine traits in male faces (Johnston and Franklin, 1993), and is likely related to the biological perception of attractiveness. Male faces and female faces diverge at puberty, making sex-respective traits especially prominent. In males, testosterone stimulates the growth of the jaw, cheekbones, brow ridges, center of the face (from the brow to the bottom of the nose), and facial hair. In females, the growth of male-centered traits is inhibited by estrogen, and estrogen has been linked to increased lip size (Thornhill and Møller, 1997). Because sexual dimorphisms increase at puberty, sexually dimorphic traits are suggested to signal sexual maturity and reproductive potential.

### Makeup and Facial Attractiveness

Revealing a sexual dimorphism in facial coloration, Nestor and Tarr (2008) found that on average, females have lighter skin than males, who are typically darker and ruddier. The researchers also found that there is a difference in facial coloration across different racial and ethnic groups. Further research has noted that faces are characterized by a typical sexually dimorphic pattern of darker features and lighter skin that varies by sex (Sinha, 2002). For example, Russell (2009) demonstrated that the difference in luminance between facial features (eyes and mouth) and skin is sexually dimorphic. Terming this difference, “facial contrast,” Russell found that increasing the contrast of the eyes and mouth in computer-manipulated faces leads to higher ratings of attractiveness for females, but lower ratings for males (Russell, 2003). Russell’s findings are consistent with historical uses of makeup by females to enhance female attractiveness by darkening the eyes and mouth relative to the surrounding skin (Corson, 2003; Russell, 2009). This normative makeup practice may work to exaggerate the sex difference in facial contrast.

In addition to its impact on facial contrast, makeup can also alter the apparent size of facial features (e.g., making eyes appear larger). Research examining the impact of makeup on perceptions of eye size demonstrated that individually, eyeliner, mascara. and eye shadow make eyes appear larger, thus increasing the sexually dimorphic trait of larger eyes among females (Matsushita et al., 2015; Morikawa et al., 2015). Importantly, however, these forms of makeup only increased perceived eye size when used independent of one another (i.e., when combined eyeliner and mascara do not make eyes appear larger). The researchers posit that the induction of visual illusions serves as one avenue by which makeup and cosmetics alter facial appearance.

### Attractiveness and Social Interaction

Through its positive effect on facial attractiveness, makeup has also been implicated in producing inflated social perceptions and more favorable social interactions. In a study directly examining how makeup affects ratings of attractiveness, competence, likeability, and trustworthiness, researchers presented participants with photos of female faces with minimal, moderate, or dramatic makeup (Etcoff et al., 2011). The researchers found that when faces were shown for 250 ms, makeup had significant positive effects on all outcomes. These results suggest that facial attractiveness has a significant positive effect on judgments of competence, likeability, and trustworthiness. Another study conducted by Klatt et al. (2016), examined the influence of different styling combinations on the evaluation of women’s leadership abilities. In presenting participants with photos of women in varied combinations of clothing (skirt/pants), jewelry (with/without jewelry), makeup, (with/without makeup) and hairstyle (loose hair/braid), the researchers found that women wearing makeup, pants, or jewelry were rated as more competent than women without makeup, wearing skirts, or not wearing jewelry. Results also indicated that the combination of loose hair and no makeup was perceived as the warmest, and overall women with loose hair were more likely to be hired than those with braids. Separate from these inflated perceptions associated with makeup, makeup has also been linked to perceptions of more unrestricted sexuality, or a willingness to engage in uncommitted sexual relationships (Osborn, 1996; Mileva et al., 2016; Batres et al., 2018).

In addition to its perceptual effects, facial attractiveness has also been found to significantly influence social interactions. Research on the interactions between mothers and their firstborn infants found that in comparison to mothers of less attractive infants, mothers of more attractive infants displayed greater affection and playfulness toward their infants (Langlois et al., 1995). With regard to the workplace, it has also been found that physically attractive men and women earn approximately 10–15 percent more than unattractive men and women. Furthermore, physically attractive individuals are expected to have more prestigious occupations than those of lesser attractiveness (Dion et al., 1972; Hamermesh and Biddle, 1993). The same study found that participants perceived attractive individuals as making more competent spouses and having better overall prospects for happy, social, and professional lives than less attractive individuals (Dion et al., 1972).

### Current Study

Although present research on facial attractiveness provides great insight on makeup’s enhancing effects, the methodology employed by most studies tests a relatively narrow set of conditions. Because many of the studies on makeup involve professional makeup artists, it is difficult to discern whether their application techniques accurately reflect those that typical women use on a day-to-day basis. Another limitation posed by several of the studies examining differential ratings of perceived competence and success is the focus on managerial and business-executive positions. While business positions are an important point of examination because of the particularly low occupation rates for females, we would argue that perceptions made in the academic setting, a much earlier point in a woman’s career, may be equally influential on their success. To address these issues, the current study seeks to advance the facial attractiveness literature through examination of the effects of makeup on facial attractiveness using different face stimuli than in previous studies (self-applied makeup in college-age participants) and examining the social implications of makeup use for women in a university setting. To accomplish the goals of the study, we collected facial stimuli through a process by which participants applied their own makeup. These stimuli were then used to evaluate the impact of makeup on perceived facial attractiveness, competence and sociosexuality.

We tested attractiveness to determine whether self-applied light or heavy was rated as more attractive compared to wearing no makeup. We were interested in looking at this as previous data have been somewhat mixed (Etcoff et al., 2011; Tagai et al., 2016). Although previous work commonly focuses on warmth and competence together, we decided to focus only on competence because our interest is in the academic setting where we believe competence is more critical for the future career success of females. Sczesny and Kühnen (2004) note that the influence of physical appearance on perceived competence is complex and involves not only gender stereotypes, but also biases based on sexual dimorphisms. We were interested in testing how ratings of sociosexuality are influenced by varying levels of makeup due to the implications of perceptions of sociosexuality on things such as sexual harassment (Kennair and Bendixen, 2012). From a practical point of view, understanding how makeup influences perceptions of attractiveness, competence, and sociosexuality may help women decide how to present themselves in different settings.

Based on previous research findings, we anticipate that the results of this project will replicate previous studies that have shown that makeup has a significant effect on perceived facial attractiveness, competence, and sociosexuality. Our more ecologically valid self-applied makeup application procedure may lead to results different from research using professional makeup artists to apply makeup. We also predict that varying levels of makeup will differ in their effects on the responses of participants across these different types of judgments.

## Experiment

In this study we compared female faces with no makeup, self-applied light makeup, and self-applied heavy makeup. Participants rated faces on attractiveness, competence, and sociosexuality so we could measure a range of traits that have been found to relate to makeup use and attractiveness. The goal was to determine if self-applied makeup leads to similar findings compared to makeup applied by a professional makeup artist (Batres et al., 2018; Etcoff et al., 2011; Osborn, 1996) or the experimenter (Killian et al., 2018). A portion of this data was presented at the Annual Meeting of the Vision Sciences Society (Aguinaldo and Peissig, 2019).

## Methods

### Materials

#### Stimuli

Undergraduate women were photographed with varying levels of makeup (no makeup, light makeup, heavy makeup) across the span of two sessions. Each subject participated in two, 30-min data collection sessions. Sessions comprised of participants being photographed with no makeup first, then either light makeup or heavy makeup. Prior to attending each session, participants were asked to bring all necessary makeup supplies for applying their own makeup. All photographs were taken using a standardized procedure, holding constant the lighting and distance of the camera (Canon EOS 700 D with EF-S 18–55 mm; Tokyo, Japan). Participants were asked to look directly at the camera with a neutral facial expression.

In the first session, the primary researcher briefly explained the study to participants before providing them with the consent form and offering to answer any questions, should they arise. Following consent, participants were verbally asked if they currently had any makeup on or if they were using any beauty enhancement products (e.g., Latisse–an eyelash growth enhancer or eyelash extensions), for the purposes of ensuring consistency among the facial stimuli collected. All participants were provided one face wipe to clean their face prior to being photographed, to ensure there was no residual makeup on their faces in the no makeup condition. The first photograph taken in the session was of participants with no makeup. Subsequently, participants were asked to apply what they would consider to be “light makeup,” or makeup that they would wear on a daily basis. After completing their makeup application participants were photographed once more.

The second session followed the same procedure as the first: participants were asked if they were currently wearing makeup, and provided a face wipe to clean their face prior to being photographed. The first photograph taken was of participants with no makeup on. Subsequently, participants were asked to apply what they would consider to be “heavy makeup,” or makeup that they would wear on a night out or special occasion. After completing their makeup application, participants were photographed once more, then given a debriefing form that provided them with further information about the experiment and the contact information of the primary investigator. We split the makeup application phase into two separate sessions to avoid issues with applying then removing makeup. We were concerned that there would be residue left from the previous makeup application and that the rubbing required for removal might lead to skin irritation or discoloration.

**FIGURE 1:**
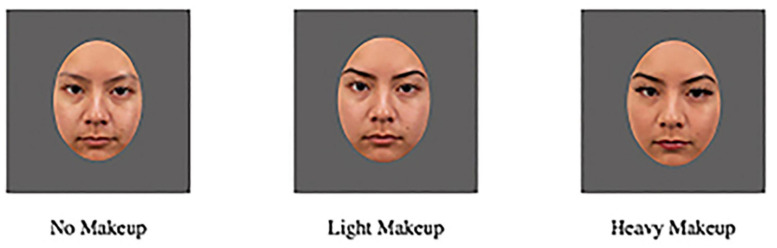
Example of Facial Stimuli (No Makeup, Light Makeup, and Heavy Makeup).

Stimuli were reviewed for completeness and picture quality, and standardized using Adobe Photoshop (standardized photographs for no makeup, light makeup, and heavy makeup applications; see Figure 1). A total of six participants were removed due to either missing the second session or unusable photographs. Unusable photographs resulted from participants not looking directly at the camera, having expressions that did not appear neutral, or images that were blurry. Thus, the final set of images contained high quality images across all conditions, resulting in a final number of 35 remaining participants. We chose to take two photographs of participants with no makeup (in both the first and second session) for consistency across sessions (we took one photo with and without makeup for each session). For this particular study we chose to use only one of the two no makeup images, to keep the number of judgments (attractiveness, competence, and sociosexuality) equal across the three makeup conditions. We chose the final single no makeup image to use for each face by visually inspecting the images and choosing whichever one appeared to have slightly better quality and head positioning, or by randomly choosing one. Similar to previous work, a uniform oval mask (1.2 inches high by 0.9 inches wide with Photoshop) was applied to the faces in order to prevent unintended effects from confounding variables such as background, hair, or face contour (Tagai et al., 2016; Killian et al., 2018). This also ensured that participants focused on the interior features of the face that were influenced by makeup, rather than external features. We decided on a final number of 35 different individuals for the face stimuli as this was a few more than our previous attractiveness study that used 30 images (Killian et al., 2018). These participants ranged in age from 18 to 27 with an average age of 19.44 (SD = 2.12). Half of the participants identified as Hispanic/Latino (*n* = 17, 48.57%), while others identified as Asian (*n* = 8, 22.86%), Pacific Islander (*n* = 3, 8.57%), Biracial/Multiracial (*n* = 3, 8.57%), White (*n* = 2, 5.71%), Other (*n* = 1, 2.86%), and one participant did not respond (*n* = 1, 2.86%).

Following the collection of facial stimuli, the faces were independently rated by another group of participants (*n* = 28) to ensure that no facial stimuli were significantly more or less attractive than any other stimuli. These participants were shown facial stimuli from the 35 different individual females and asked to rate the faces presented on facial attractiveness using a 1–7 Likert scale, with 1 being very unattractive and 7 being very attractive. Only the no makeup version of the faces was shown for this rating. Results from the rating study revealed that participant ratings for each of the facial stimuli were within two standard deviations of the overall mean attractiveness ratings (*M* = 3.56, SD = 0.66). Given the absence of any minor or major outliers, all facial stimuli were used for the experiment.

We quantitatively measured for contrast differences in the no makeup, light makeup, and heavy makeup conditions. Our measurement was based on that used by Russell (2003, 2009), by using the Michelson contrast formula to calculate a facial contrast value (C_F_) for the 35 faces in each of the three conditions. We found that the mean *C*_F_ value was lowest for faces with no makeup (*M* = 0.213), light makeup faces had a slightly higher mean *C*_F_ value (*M* = 0.227), and the heavy makeup faces had the highest mean *C*_F_ value (*M* = 0.273). Paired *t*-tests indicated that the facial contrast value difference between the heavy makeup and no makeup was significant (*t*(102) = −4.26; *p* < 0.0001). The difference between the heavy makeup and the light makeup images was also statistically significant (*t*(102) = −3.23; *p* = 0.0016). However, the facial contrast value difference between the light makeup and no makeup images was not significant (*t*(102) = −1.02; *p* > 0.05).

#### Experiment

The computer-based experiment was created and administered using SuperLab 5 software^1^. The program was run on three 21-inch, 2013 iMacs (Apple Incorporated, Cupertino, CA, United States).

#### Demographics Survey

The survey was administered through the online survey platform, Qualtrics^2^. Survey questions gathered demographic information and assessed positive and negative attitudes toward makeup use.

### Experiment Participants

The experiment was run several months after the face stimuli were collected, reducing the probability that participants would be familiar with individuals in the face stimulus set. In addition, both groups of participants were recruited primarily from sections of the introduction to psychology course, which are mostly first year students and include both majors and non-majors. Thus, the participants were very unlikely to have encountered the students from whom the faces were collected. A total of 69 students were recruited through the CSUF Psychology Department human subject pool. Individuals were awarded course credit for their participation. Participants were predominantly female (*n* = 44, 64%) with a smaller number of males (*n* = 22, 32%), one non-binary, and two participants who did not report their gender. Participants ranged in age from 18 to 53 with an average age of 19.97 (SD = 4.55). A third of participants identified as Hispanic/Latino (*n* = 24, 34.78%), while another third identified as Asian/Pacific Islander (*n* = 24, 34.78%). The remainder reported themselves as White/European (*n* = 10, 14.49%), Biracial/Multiracial (*n* = 6, 8.70%), Middle Eastern (*n* = 4, 5.80%), and Black (*n* = 1, 1.45%).

### Procedure

Subjects participated in a SuperLab experiment in which they were presented with the standardized facial stimuli. They responded using an RB-840 response keypad (Cedrus Corporation, San Pedro, CA, United States), which includes eight response buttons; only seven buttons were used in this experiment. The participants were shown the labeled-response keypad specific to their condition, pressed any key to proceed, and then viewed a 500 ms fixation cross, followed by the face image, along with an image of the keypad with the forced choice responses labeled (1–7 and what each response corresponded to depending on condition); the keypad image appeared below the face image. Following Etcoff et al. (2011), participants were allowed to view the face image and keypad response image until they responded. Each of the 69 participants in the experiment viewed the 35 individuals in three different forms: no makeup, light makeup, and heavy makeup. These 105 stimuli were completely randomized within each test session. Because students who participated in the experiment came from the same university as those who were used as stimuli, on their completion of the experiment, those who rated the stimuli were asked verbally if they personally knew any of the students photographed. No participants reported knowing any of the individuals photographed as stimuli. Participants were randomly assigned to one of three groups (i.e., Facial Attractiveness, Competence, Sociosexuality), indicating which face judgment task they would do. Numbers of participants differed slightly across groups because participants were recruited until the deadline for data collection for the semester. In the end we were left with slightly unequal numbers across groups (24/22/23). We decided to keep all participants rather than discard any data.

#### Facial Attractiveness

Twenty-four participants in the facial attractiveness group were asked to rate the faces presented on facial attractiveness using a 1–7 Likert scale with 1 being very unattractive and 7 being very attractive.

#### Competence

Twenty-two participants in the competence group were asked to rate the faces presented on perceived competence using a 1–7 Likert scale with 1 being very incompetent and 7 being very competent.

#### Sociosexuality

Twenty-three participants in the sociosexuality group were asked to rate the faces presented on their sociosexuality. They rated how likely they believed the person would be to have casual sex, using a 1–7 Likert scale with 1 being very unlikely and 7 being very likely.

## Results

Separate cross-classified multilevel models were constructed to determine the predictive value of Makeup Application (no makeup, light makeup, heavy makeup) for Attractiveness, Competence, and Sociosexuality.

### Attractiveness

A cross-classified multilevel model predicting ratings of Attractiveness by Makeup Application (no makeup, light makeup, heavy makeup) was created using the Heavy Makeup stimuli as the reference group. Thus, coefficients in the No Makeup and Light Makeup stimuli groups compared attractiveness ratings to those in the Heavy Makeup stimuli groups. The variability in attractiveness ratings across participants and stimuli as well as the variability in the effect of makeup on attractiveness ratings across participants and stimuli were included in the model as random effects. The item Makeup had a significant impact on the Attractiveness ratings of the participants, χ^2^(6) = 1865.80, *p* < 0.001. Participants’ predicted Attractiveness ratings are equal to 3.53 + 0.25 (Makeup Application). Participants’ average Attractiveness ratings increased by 0.25 for each increase in Makeup Application (Table 1). Different from other statistical approaches that would only use the average of participants’ attractiveness ratings across stimuli, our cross-classified multilevel model’s consideration of variance across participants and stimuli produces a more accurate measure and subsequent interpretation of makeup’s effect on participants’ perceptions of attractiveness.

**TABLE 1:** Parameter estimates for multilevel model predicting Attractiveness ratings from Makeup Application.

**Parameter**	**Estimate**	**SE**	***df***
*Fixed Effects*			
Intercept	3.5310***	0.1978	23
Makeup	0.2490***	0.05446	23
*Random Effects*			
Participant			
τ_00_	0.7550	0.2287	
τ_10_	0.009090	0.03888	
τ_11_	0.03231	0.1318	
Stimuli			
τ_00_	0.2386	0.06493	
τ_10_	–0.01785	0.02189	
τ_11_	0.03867	0.01363	
σ^2^	0.9087	0.02514	

***p < 0.001. Participant τ_00_ = Variance in intercepts between participants. τ_10_ = Correlation between intercepts and slopes. τ_11_ = Variance in the effect of Makeup Application on Attractiveness ratings. Stimuli τ_00_ = Variance in intercepts between stimuli. τ_10_ = Correlation between intercepts and slopes. τ_11_ = Variance in the effect of Makeup Application on Attractiveness ratings. σ^2^ or Residual = Variance between participants/stimuli.

**FIGURE 2:**
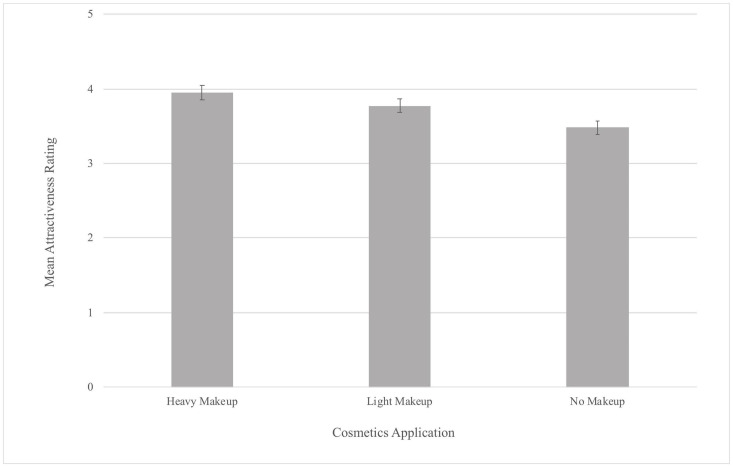
Mean Attractiveness Ratings by Makeup Application (error bars represent 95% confidence intervals).

Our Tukey’s *post hoc* analysis revealed that participant’s Attractiveness ratings were significantly higher for the heavy makeup application (*M* = 3.95) than for the light makeup application (*M* = 3.77, *b* = −0.19, *p* < 0.05). Additionally, participant’s Attractiveness ratings were significantly higher for the light makeup (*b* = −0.3, *p* < 0.001) and heavy makeup applications (*b* = −0.49, *p* < 0.001) than the no makeup application (*M* = 3.48; see Figure 2). A *post hoc* power analysis indicated that the power for this attractiveness experiment was 0.74.

### Competence

A cross-classified multilevel model predicting ratings of Competence by Makeup Application (no makeup, light makeup, heavy makeup) was created using the Heavy Makeup stimuli as the reference group. Thus, coefficients in the No Makeup and Light Makeup stimuli groups compared competence ratings to those in the Heavy Makeup stimuli groups. The variability in competence ratings across participants and stimuli as well as the variability in the effect of makeup on competence ratings across participants and stimuli were included in the model as random effects. The item Makeup had a significant impact on the Competence ratings of the participants, χ^2^(6) = 1161.42, *p* < 0.001. Participants’ predicted Competence ratings are equal to 4.17 + 0.08 (Makeup Application). Participants’ average Competence ratings increased by 0.08 for each increase in Makeup Application (Table 2). Different from other statistical approaches that would only use the average of participants’ competence ratings across stimuli, our cross-classified multilevel model’s consideration of variance across participants and stimuli produces a more accurate measure and subsequent interpretation of makeup’s effect on participants’ perceptions of competence.

**TABLE 2:** Parameter estimates for multilevel model predicting Competence ratings from Makeup Application.

**Parameter**	**Estimate**	**SE**	***df***
*Fixed Effects*			
Intercept	4.1686***	0.2031	21
Makeup	0.07727***	0.03868	21
*Random Effects*			
Participant			
τ_00_	0.7395	0.2373	
τ_10_	0.02533	0.02652	
τ_11_	0.001545	0.005924	
Stimuli			
τ_00_	0.2204	0.06478	
τ_10_	−0.01176	0.02095	
τ_11_	0.02194	0.01213	
σ^2^	1.2301	0.03711	

***p < 0.001. Participant τ_00_ = Variance in intercepts between participants. τ_10_ = Correlation between intercepts and slopes. τ_11_ = Variance in the effect of Makeup Application on Competence ratings. Stimuli τ_00_ = Variance in intercepts between stimuli. τ_10_ = Correlation between intercepts and slopes. τ_11_ = Variance in the effect of Makeup Application on Competence ratings. σ^2^ or Residual = Variance between participants/stimuli.

**FIGURE 3:**
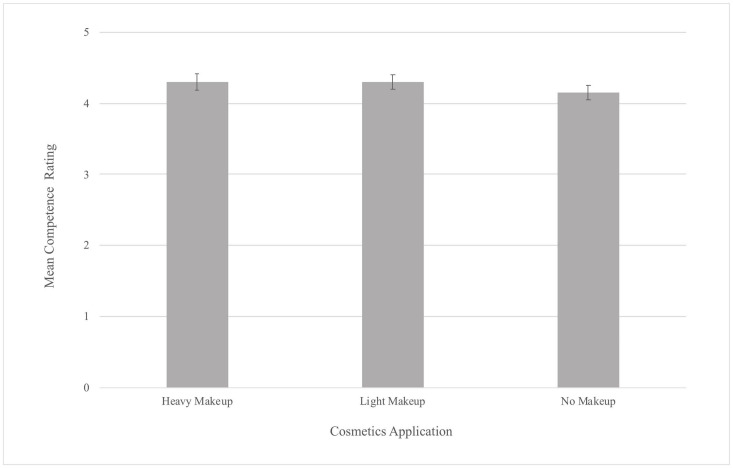
Mean Competence Ratings by Makeup Application (error bars represent 95% confidence intervals).

Our Tukey’s *post hoc* analysis revealed that participant’s Competence ratings were significantly higher for the light makeup (*M* = 4.29, *b* = −0.15, *p* < 0.05) and heavy makeup applications (*M* = 4.3, *b* = −0.15, *p* < 0.05) than for the no makeup application (*M* = 4.14; see Figure 3). A *post hoc* power analysis indicated that the power for this attractiveness experiment was 0.70.

### Sociosexuality

A cross-classified multilevel model predicting ratings of Sociosexuality by Makeup Application (no makeup, light makeup, heavy makeup) was created using the Heavy Makeup stimuli as the reference group. Thus, coefficients in the No Makeup and Light Makeup stimuli groups compared sociosexuality ratings to those in the Heavy Makeup stimuli groups. The variability in sociosexuality ratings across participants and stimuli as well as the variability in the effect of makeup on sociosexuality ratings across participants and stimuli were included in the model as random effects. The item Makeup had a significant impact on the Sociosexuality ratings of the participants, χ^2^(6) = 828.89, *p* < 0.001. Participants’ predicted Sociosexuality ratings are equal to 3.87 + 0.52 (Makeup Application). Participants’ average Sociosexuality ratings increased by 0.52 for each increase in Makeup Application (Table 3). Different from other statistical approaches that would only use the average of participants’ sociosexuality ratings across stimuli, our cross-classified multilevel model’s consideration of variance across participants and stimuli produces a more accurate measure and subsequent interpretation of makeup’s effect on participants’ perceptions of sociosexuality.

**TABLE 3:** Parameter estimates for multilevel model predicting Sociosexuality ratings from Makeup Application.

**Parameter**	**Estimate**	**SE**	***df***
*Fixed Effects*			
Intercept	3.3872***	0.1914	22
Makeup	0.5205***	0.07248	22
*Random Effects*			
Participant			
τ_00_	0.6996	0.2211	
τ_10_	−0.1200	0.06521	
τ_11_	0.08050	0.03038	
Stimuli			
τ_00_	0.1665	0.05284	
τ_10_	−0.00594	0.02082	
τ_11_	0.03055	0.01491	
σ^2^	1.4167	0.04177	

***p < 0.001. Participant τ_00_ = Variance in intercepts between participants. τ_10_ = Correlation between intercepts and slopes. τ_11_ = Variance in the effect of Makeup Application on Sociosexuality ratings. Stimuli τ_00_ = Variance in intercepts between stimuli. τ_10_ = Correlation between intercepts and slopes. τ_11_ = Variance in the effect of Makeup Application on Sociosexuality ratings. σ^2^ or Residual = Variance between participants/stimuli.

**FIGURE 4:**
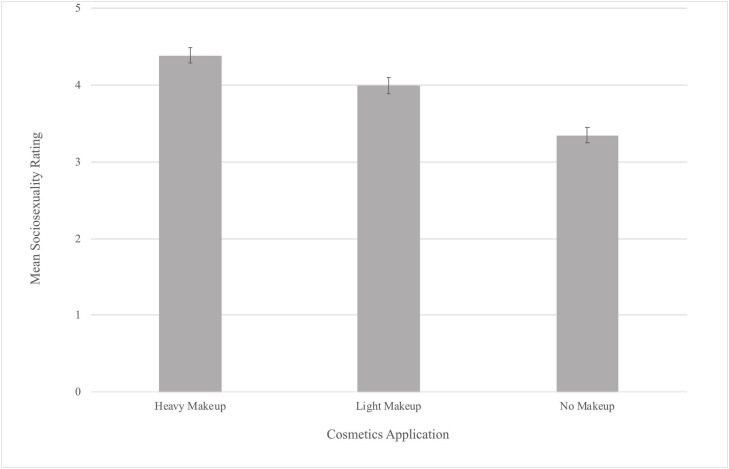
Mean Sociosexuality Ratings by Makeup Application (error bars represent 95% confidence intervals).

Our Tukey’s *post hoc* analysis revealed that participant’s Sociosexuality ratings were significantly higher for the heavy makeup application (*M* = 4.39) than for the light makeup application (*M* = 3.99, *b* = −0.39, *p* < 0.001). Additionally, participant’s Sociosexuality ratings were significantly higher for the light makeup (*b* = −0.65, *p* < 0.001) and heavy makeup applications (*b* = −1.04, *p* < 0.001) than the no makeup application (*M* = 3.34; see Figure 4). A *post hoc* power analysis indicated that the power for this attractiveness experiment was 0.72.

## Discussion

### Summary of Findings

In our investigation of makeup’s influence on perceived facial attractiveness, competence, and sociosexuality, we found that, as predicted, makeup had a significant effect on ratings for all three measures. Additionally, we found that no makeup, light makeup, and heavy makeup application significantly differed in their effects on perceived facial attractiveness, and sociosexuality. For competence judgments, we found that both the light and heavy makeup applications differed from the no makeup condition, but light and heavy makeup application did not differ from each other.

### Facial Attractiveness

Faces with light makeup were rated significantly more attractive than faces with no makeup and faces with heavy makeup were rated significantly more attractive than both no makeup and light makeup faces. Overall, faces with heavy makeup were rated as most attractive.

These results are consistent with work from Etcoff et al. (2011) which demonstrated higher attractiveness and competence ratings for heavy (glamorous and professional) makeup compared to light (natural) makeup. However, the findings differ from other research in which faces with light makeup yielded higher attractiveness ratings than faces with no makeup or heavy makeup (Tagai et al., 2016). While these contrasting findings might suggest differences in participants’ perceptions of attractiveness, we instead posit that the different results may be due to distinct methodological techniques used in how researchers created their facial stimuli. In both previous studies, light makeup and heavy makeup facial stimuli were created using professional makeup artists. Despite both research teams using professional makeup artists, they reported different results with seemingly equivalent makeup application conditions. It might be that the makeup artists differed in the amount of makeup they applied, or the particular techniques use. Although having a professional makeup artist apply the makeup allows for the standardization of makeup application across a set of facial stimuli, it may not accurately reflect the makeup that typical women use on a day-to-day basis. We consider our procedure of having participants self-apply light and heavy makeup for facial stimuli more ecologically valid, and a strength of our study (although the variability among individual makeup application may also be considered a weakness when compared to the consistency offered by professional makeup artists). Our finding that heavy makeup faces yield the highest attractiveness ratings more accurately reflects self-applied makeup in everyday life. Thus, our data suggest that when women apply their own makeup, rather than have their makeup applied by a professional makeup artist, heavy makeup is considered more attractive than light makeup. Another possibility is that in some cases the light makeup applied by professional makeup artists more closely resembles self-applied heavy makeup. To test this possibility and investigate other differences between makeup application among professional makeup artists and average makeup wearers, however, more research is needed, ideally using quantitative measures of makeup to compare across different types of makeup application.

### Competence

While faces with light makeup and heavy makeup each yielded significantly higher competence ratings than faces with no makeup, they did not significantly differ from each other. In their work examining the effects of makeup on perceptions of competence, Klatt et al. (2016) found that faces with makeup were rated higher on competence than faces without makeup; however, they did not vary makeup application (i.e., light vs. heavy makeup). Although the degree of makeup application affects perception of attractiveness and sociosexuality, there may be no such effect on perceptions of competence. In the case of competence, Tsankova and Kappas (2016) explain that skin smoothing makeup may indirectly impact perception through signaling an attention to detail and subsequently greater potential competence. Our work extends other research on the effects of makeup on perceived competence through the use of a college-aged sample. As previously mentioned, much of the current work on this topic has examined this relationship in business-level settings, using middle-aged women as facial stimuli (Klatt et al., 2016). Our work demonstrates a similar effect at an earlier stage in women’s careers.

### Sociosexuality

Faces with light makeup received significantly higher sociosexuality ratings (rated as significantly more likely to have “casual” sex with multiple partners) than faces with no makeup and faces with heavy makeup received significantly higher sociosexuality ratings than both no makeup and light makeup faces. Overall, faces with heavy makeup were rated as the most likely to have “casual” sex with multiple partners. These results extend recent work, in which researchers found that faces with makeup were perceived as more sociosexual than the same faces without makeup (Batres et al., 2018). Previous research has suggested that this increase in perceived sociosexuality may be due to makeup serving as a potential cue to availability (Guéguen, 2008). Our findings suggest that in addition to differences in perceived sociosexuality between no makeup and makeup faces, faces with heavy makeup are perceived as more sociosexual than faces with light makeup. The amount of makeup may be perceived as signal of sociosexual behavior that may or may not be related to the actual wearer’s intentions.

## Conclusion

We found support for our proposal that makeup would have significant effects on perceptions of facial attractiveness, competence, and sociosexuality. Ratings of facial attractiveness and sociosexuality were highest for faces with heavy makeup. Ratings of competence for faces with light makeup and heavy makeup were both higher than ratings for faces with no makeup, but there were no differences between faces with light makeup and heavy makeup. Our results suggest that self-applied heavy makeup will provide more positive results for attractiveness judgments compared to self-applied light makeup, a finding that is counter to the advice often given in popular media. It is usually suggested that “less is more” and that lighter makeup is more attractive (Doyle, 2019; Almanza and Young, 2020). Our data show that people preferred the look of a heavier makeup application, at least in the conditions we tested. In contrast, the heavier makeup also led to perceptions of greater sociosexuality, but did not increase perceptions of competence. Research showing greater potential for harassment for those rated as having higher sociosexuality (Kennair and Bendixen, 2012) suggest that wearing heavy makeup may also have negative consequences. Thus, this study presents a more complex picture of makeup use for women, in which the amount of makeup a woman chooses to wear affects a variety of visual and social perceptions.

This study significantly expands our knowledge of how makeup use affects perceptions of others. Through advancing this literature, we are able to increase the societal understanding of why makeup influences social perception of women. A better understanding of these issues may help us increase well-being and success.

## Data Availability Statement

The raw data supporting the conclusions of this article will be made available by the authors, without undue reservation.

## Ethics Statement

The studies involving human participants were reviewed and approved by California State University, Fullerton Institutional Review Board. The patients/participants provided their written informed consent to participate in this study. Written informed consent was obtained from the individual(s) for the publication of any potentially identifiable images or data included in this article.

## Author Contributions

EA developed the research idea, created the study, and collected the data, with help from JP. EA was also responsible for writing the manuscript, with input and revisions contributed by JP. All authors contributed to the article and approved the submitted version.

## Conflict of Interest

The authors declare that the research was conducted in the absence of any commercial or financial relationships that could be construed as a potential conflict of interest.

## Footnotes

1 https://www.cedrus.com/superlab/

2 https://www.qualtrics.com/
